# Cognitive impairment reversed by cinacalcet administration in primary hyperparathyroidism

**DOI:** 10.1007/s42000-021-00292-4

**Published:** 2021-04-21

**Authors:** J. G. Timmons, R. Manners, M. Bailey, C. McDougall

**Affiliations:** 1 Institute of Cardiovascular and Medical Sciences, University of Glasgow, Glasgow, UK; 2 Department of Diabetes, Endocrinology and Clinical Pharmacology, Glasgow Royal Infirmary, Glasgow, UK; 3 Department of Care of the Elderly, University Hospital Hairmyres, East Kilbride, UK; 4 Department of Diabetes and Endocrinology, University Hospital Hairmyres, East Kilbride, UK

**Keywords:** Primary hyperparathyroidism, Cinacalcet, Hypercalcemia, Cognitive impairment

## Abstract

Primary hyperparathyroidism (pHPT) is a common endocrine disorder. Often serum calcium is minimally elevated with few symptoms. In elderly patients with multiple co-morbidities, the decision to “watch and wait” is often most clinically appropriate as operative intervention is associated with high peri-operative risk. We present an elderly patient with mild hypercalcemia secondary to primary hyperparathyroidism. The clinical decision was initially to watch and wait. The patient subsequently developed cognitive impairment and was diagnosed with mixed Alzheimer’s disease/vascular dementia. She became dependent for all care and housebound. A therapeutic trial of cinacalcet was commenced following a further acute rise in serum calcium. Significant reversal of her functional and cognitive deficit occurred. She was no longer fully dependent. Mini mental state examination (MMSE) improved from 8/30 to 21/30. In vulnerable neural systems, even mild elevation in serum calcium may have a profound effect on cognition and function. We propose a therapeutic trial of cinacalcet in such patients.

## Background

Hypercalcemia is a common biochemical abnormality encountered in clinical practice [1, 2]. While important causes such as malignancy and myeloma should be excluded, in the context of an inappropriately normal or elevated parathyroid hormone (PTH) level, a diagnosis of primary hyperparathyroidism is made [1]. In people who are otherwise fit, operative intervention offers cure [1, 2]. Primary hyperparathyroidism frequently presents in the elderly who are multimorbid and therefore surgical intervention is not possible [3]. In these patients, if they are relatively asymptomatic, an active surveillance approach is adopted with intermittent monitoring of serum calcium [3]. We present a case of a patient with mixed dementia, marked cognitive and functional decline, and co-morbid primary hyperparathyroidism. The patient was unfit for surgical management. An acute rise in serum calcium to 3.23 mmol/L (2.20–2.60 mmol/L) was incidentally found on admission bloods following a fall. After initial fluid resuscitation, cinacalcet was commenced to prevent further acute serum calcium rises. Unexpectedly, there was a significant cognitive and functional improvement over a 6-month period following commencement of cinacalcet.

## Clinical case

Mrs M. an 87-year-old woman with a background of mixed vascular and Alzheimer’s dementia was referred to our service while an inpatient following a fall. The patient had a past medical history of type 2 diabetes mellitus, osteoporosis, hypothyroidism, ischemic heart disease, and primary hyperparathyroidism (pHPT) — under observation at a neighbourhood health center.

Mrs M. had biochemical evidence of pHPT for several years from her GP records. On initial referral to another endocrine service, PTH was 9.9 pmol/L (1.6–6.9 pmol/L) with a corresponding adjusted calcium of 2.99 mmol/L. Random urine calcium to creatinine ratio was raised at 0.71 mmol/mol. Renal function was normal. Vitamin D levels were insufficient at 25 nmol/L (> 50 nmol/L) and myeloma screen was negative. Mrs M. was independently mobile with a stick and had no overt cognitive impairment. The patient had asymptomatic hypercalcemia. Vitamin D was adequately replaced, and Mrs M. was recommended for endocrinological follow-up.

However, in the interim, Mrs M. had a period of cognitive decline and worsening mobility with falls. She was referred to old age psychiatry. CT brain demonstrated age-related cerebral atrophy with small vessel disease. Mini mental state examination (MMSE) was 9/30 and functional assessment with occupational therapy demonstrated significant functional impairment. Alzheimer’s and vascular mixed dementia was diagnosed. Her cognitive condition worsened, and Mrs M. required a full package of care with carers attending 4 times daily. She became housebound and incontinent.

Mrs. M received acute medical admission to our hospital following a fall at home. Her calcium was found to be 3.23 mmol/L. PTH was elevated at 11.4 pmol/L. Vitamin D was 55 nmol/L. She was hydrated with IV fluids to urgently lower her calcium. Endocrinological guidance was sought and cinacalcet 30 mg twice daily was commenced.

## Outcome

In the clinic 6 months later, adjusted calcium had fallen to 2.30 mmol/L with a corresponding PTH of 4.0 pmol/L. There had been a marked improvement in her functional status since commencing cinacalcet. Mrs M. was now independently dressing and washing, and her daughter reported that she was doing her own housework again. Her package of care had been significantly reduced. Mrs M. was no longer incontinent. There had been no further admissions with falls. Her family felt that her cognitive state was vastly improved. Her daughter reported that she felt her mother no longer had any significant appreciable cognitive impairment in day-to-day interaction. No other drugs for dementia had been commenced over the intervening period. Repeat MMSE was performed with an improvement in her score to 21/30 from 9/30.

## Discussion

Primary hyperparathyroidism is characterized by over production of PTH [1, 2, 4]. It is characterized biochemically by elevated adjusted serum calcium with an inappropriately non-suppressed or frankly elevated PTH [2]. This leads to increased bone calcium resorption, increased intestinal calcium uptake, and augmented tubular resorption of calcium. Furthermore, PTH acts to increase conversion of vitamin D to its active form [4]. In approximately 80% of cases, a single underlying benign parathyroid adenoma is responsible. In 15%, parathyroid hyperplasia of multiple glands is present. The remaining 5% is composed of both multiple adenomas and very rarely parathyroid carcinoma [2, 4]. In an elderly and frail population with multimorbidity, the management of pHPT can be challenging [1, 5]. Definitive management in the form of parathyroidectomy is often curative in the context of a single benign parathyroid adenoma with excess PTH secretion [1]. Surgical intervention however is often precluded due to significant co-morbidity and resultant anesthetic risk in older people. In this context and with mild hypercalcemia, an approach of active surveillance is often adopted by endocrinologists as was the case with this patient [1, 6]. This is particularly true when the patient is asymptomatic and hypercalcemia is detected only incidentally. This is true in up to 80% of cases [1, 2, 5]. When symptoms are present, they vary from mild subtle symptomatology to more severe symptoms likely to lead to presentation for investigation. Urinary frequency and thirst from the osmotic effect of high calcium may be present. Patients may complain of constipation and abdominal discomfort. More severe symptoms associated with nephrolithiasis or bone disease may however be the presenting feature. [4]

Hypercalcemia is known to be associated with numerous neurocognitive abnormalities ranging from subtle cognitive and mood changes to overt cognitive impairment with dementia [5]. Low mood and anxiety may precede more significant neurocognitive effects of hypercalcemia. Often this presents in a subacute manner over months to years adding further diagnostic difficulty. The underlying mechanisms leading to the neuropsychiatric features of hypercalcemia are however complex and still incompletely understood [2, 4].

Cinacalcet is a calcimimetic agent. It acts on the calcium sensing receptor (CasR) through allosteric activation [7]. This is a G-protein coupled receptor which is expressed on the parathyroid cell surface and in several other tissues. In normal physiology, as extracellular calcium levels rise, calcium activates the CasR leading to a cascade of intracellular actions with the ultimate effect of reducing PTH secretion [8]. Cinacalcet binds to the CasR leading to enhanced responsiveness to extracellular calcium. This reduces PTH secretion and therefore serum calcium levels. It is low cost and well tolerated by most patients. Cinacalcet is effective in reducing serum calcium and therefore reducing the symptoms associated with this. When used in the long term, it is effective at maintaining reasonably stable calcium levels [3, 7]. In this case, it was only following an acute hypercalcemic crisis and hospital admission that definitive cinacalcet therapy was commenced.

While calcium can lead to significant neurocognitive impairment alone, it is likely that in the present case, characterized by neural system vulnerability underlying cerebrovascular disease, Alzheimer’s disease and dementia, that elevated serum calcium levels had a disproportionate impact on this patient’s neurofunctional status. The impact this subsequently had on both the patient and her extended family was profound. Significant social care input was required with the attendant resource and financial implications. Although there was a significant numerical improvement in MMSE after cinacalcet administration, it is the gain in functional status that was arguably the most significant benefit to the patient and her family.

## Conclusion

Significant functional and cognitive impairment can occur with elevated serum calcium levels in primary hyperparathyroidism. In patients who additionally have vulnerable neural systems secondary to advanced age, underlying cerebrovascular disease, and dementia, this neurological deficit may be more clinically and functionally profound. Given the high tolerability, affordability, and ongoing beneficial effects on serum calcium, a therapeutic trial of cinacalcet should be considered in people presenting with cognitive impairment and elevated serum calcium who are unfit for definitive surgical management.
